# Extended Cognitive Load Induces Fast Neural Responses Leading to Commission Errors

**DOI:** 10.1523/ENEURO.0354-24.2024

**Published:** 2025-02-06

**Authors:** Fabio Taddeini, Giulia Avvenuti, Alberto Arturo Vergani, Jacopo Carpaneto, Francesca Setti, Damiana Bergamo, Linda Fiorini, Pietro Pietrini, Emiliano Ricciardi, Giulio Bernardi, Alberto Mazzoni

**Affiliations:** ^1^The Biorobotics Institute, Scuola Superiore Sant’Anna, Pontedera 56025, Italy; ^2^Department of Excellence for Robotics and AI, Scuola Superiore Sant’Anna, Pisa 56127, Italy; ^3^School of Advanced Studies, Center for Neuroscience, University of Camerino, Camerino 62032, Italy; ^4^MoMiLab Research Unit, IMT School for Advanced Studies, Lucca 55100, Italy

**Keywords:** cognitive load, event-related potentials, Go/NoGo task, hd-EEG, response inhibition

## Abstract

Extended performance of cognitively demanding tasks induces cognitive fatigue manifested with an overall deterioration of behavioral performance. In particular, long practice with tasks requiring impulse control is typically followed by a decrease in self-control efficiency, leading to performance instability. Here, we show that this is due to changes in activation modalities of key task-related areas occurring if these areas previously underwent intensive use. We investigated in 25 healthy adults the effects of extended practice with high cognitive demand (HCD) tasks on a Go-No Go task and the underlying electroencephalographic (EEG) activity. We compared these effects with those induced by practice with similar, but low cognitive demand (LCD) tasks. HCD tasks were followed by an increase in response inhibition failures. These were correlated with the appearance of a distinct neural signature on fast response trials, characterized by lower levels of beta ([13–30] Hz) EEG activity in the prestimulus period, and by a lack of EEG markers of preresponse processing in frontal areas. Moreover, HCD tasks were followed by a decrease in N200 during correct withholds while LCD tasks were followed instead by a lesser fraction of hits and a decrease in P300, suggesting a decrease in engagement. Overall, these results show that exertion of cognitive control determines the appearance of two distinct modalities of response with different processing speeds, associated with distinct underlying neural activity.

## Significance Statement

Extended cognitive load leads to alterations in behavior, but the underlying alterations in cortical activity are far from being understood. When we compared the performance in a Go/NoGo test before and after a battery of tasks requiring high cognitive control, we found an increase in commission errors associated with an increase in fast automatic responses. EEG signals of these responses displayed a lack of cortical markers of preresponse processing. Tasks requiring only low cognitive control were followed instead by an increase in miss errors, likely related to a decrease in engagement. Extended cognitive load leads then to the appearance of two distinct response modalities, driven by distinct neural activities.

## Introduction

Extended involvement in cognitive tasks leads to a deterioration of behavioral performance that is typically reverted after a period of rest or sleep (Müller and Apps, 2019; Tran et al., 2020). This particular state, commonly indicated as mental or cognitive fatigue, is frequently observed in daily life activities. The specific manifestations of cognitive fatigue may vary depending on the context and task at hand but may generally include changes in reaction time with impulsive or sluggish responses and reduced behavioral accuracy and/or precision (i.e., increased response variability). Since cognitive fatigue may substantially increase the risk of accidents or antisocial behaviors, substantial efforts have been undertaken to characterize its behavioral, functional, and physiological bases.

Response inhibition, one of the so-called executive functions, involves being able to control one's behavior to override a strong impulse and select the more appropriate or needed behavior (Diamond, 2013). Under conditions of cognitive fatigue, individuals have been shown to fail more often at suppressing an impulsive or automatic response (commission error), while reaction times may mainly increase or decrease depending on the specific task (Kato et al., 2009; Möckel et al., 2015). Another common observation is that of an increased response instability, especially manifested with increased variability in reaction times even in the absence of obvious errors or lapses (Wang et al., 2014).

These behavioral changes are accompanied by detectable changes in brain activity, and especially in the so-called event-related potentials (ERPs) computed from electroencephalographic (EEG) recordings. Two ERP components modulated by cognitive fatigue are the N200 and P300 components, a negative and a positive EEG-signal deflection peaking ∼200 and 300 ms after stimulus onset, respectively. The N200 component is thought to be generated in the frontal cortices, presumably within the mid-cingulate cortex and ventral and dorsolateral prefrontal cortex (Lavric et al., 2004; Wessel, 2012) and is mostly associated with novelty (Wessel, 2012) and conflict monitoring (Lavric et al., 2004; Folstein and Van Petten, 2008). The P300 component, sometimes divided into an anterior frontocentral component (P300a) and a posterior parietal component (P300b), is assumed instead to mainly reflect attention allocation and response selection (Donchin and Coles, 1998; Friedman et al., 2001; Schmidt-Kassow et al., 2009; Albert et al., 2013; Strobel et al., 2015; Verleger, 2020). Previous work showed that the P300 amplitude decreases after extended practice with tasks requiring the exertion of response inhibition, while changes in N200 amplitude appear largely inconsistent across studies (Boksem et al., 2006; Kato et al., 2009; Möckel et al., 2015). A response-locked ERP negativity related to commission errors, commonly indicated as error-related negativity (ERN), also appeared to decrease in amplitude after extended task practice (Lorist et al., 2005; Boksem et al., 2006).

Interestingly, while the behavioral instability observed in conditions of cognitive fatigue seems to reflect a fluctuating, stochastic process (Gunzelmann et al., 2011), previous work mainly treated behavioral and the associated brain activity changes as a relatively uniform phenomenon. Brain activity changes were commonly measured by comparing the average across all trials with correct or incorrect outcomes across fatigued and rested (or less fatigued) conditions. Here, we hypothesized that behavioral instability resulting from cognitive fatigue may reflect the appearance of distinctive events associated with specific electrophysiological correlates. To test these hypotheses, we investigated the behavioral and physiological effects of extended practice with tasks requiring the exertion of response inhibition functions and compared such effects with those induced by practice with identical tasks not requiring control of impulses. We also analyzed relative variations in response characteristics in the two experimental conditions to identify potential markers of behavioral instability and their electrophysiological correlates.

## Materials and Methods

### Participants

Twenty-six healthy adults (age range = 21–31 years, mean ± SD = 26.2 ± 2.5 years, 16 females, all right-handed) were included in the study. Potential volunteers underwent a preliminary interview to exclude any clinical, neurological, or psychiatric conditions potentially affecting brain function and behavior. Additional exclusion criteria included the absence of relevant sleep-related issues (Pittsburgh Sleep Quality Index; score > 10; Buysse et al., 1989), excessive daytime sleepiness (Epworth Sleepiness Scale; score > 10; Johns, 1991), and extreme chronotypes (Morningness-Eveningness Questionnaire; score > 70 or score < 30; Horne & Ostberg, 1976). Participants were asked to maintain a regular sleep–wake schedule for at least 1 week before each experiment. Compliance was verified by wrist-worn actigraphy (MotionWatch 8, CamTech). The study was conducted under a protocol defined in accordance with the ethical standards of the 2013 Declaration of Helsinki and approved by the Local Ethical Committee. Written informed consent was obtained from all participants.

### Experimental design

All participants completed a training session and two experimental sessions in which high-density electroencephalographic activity (EEG; 64 electrodes; EGI) and behavioral data were recorded. The time window of each session was kept fixed to avoid possible confounding factors related to time-of-day effects or the influence of interindividual differences in daily activities (e.g., work-related fatigue). In particular, the training session was performed on Friday morning from 9:30 A.M. to 11:30 A.M., while the two experimental sessions took place on the subsequent Monday and Tuesday from 8:30 A.M. to 1:30 P.M.

The training session included the completion of two computerized psychometric questionnaires assessing impulsiveness (Barratt Impulsiveness Scale; Fossati et al., 2001; Stanford et al., 2009) and aggressiveness (Buss-Perry Aggression Questionnaire; Buss and Perry, 1992; Fossati et al., 2003) and a practice and calibration session with a classical response inhibition task (Go/NoGo, see below; Bernardi et al., 2015).

Each experimental session began with the hd-EEG cap preparation followed by a baseline test block lasting ∼15 min (BL). This test block comprised the completion of a set of Likert scales (1–9) assessing subjective alertness, sleepiness, perceived effort, mood, and motivation, resting-state EEG activity recordings, and a computerized Go/NoGo task. Then, participants completed two ∼45 min task blocks involving high (HCD) or low (LCD) cognitive control demands. Each task block included three ∼15 min tasks requiring (or not) the exertion of self-control (see below). Test blocks (T1 and T2) identical to the baseline were repeated after each task block. The two experimental sessions were completed in a pseudorandom, counterbalanced order.

To ensure signal quality during EEG recordings, electrode impedance was checked at the beginning of each test block (BL, T1, T2) and kept below 50 KΩ.

### Go/NoGo task

During each test block, participants completed two runs of a classical Go/NoGo task (“XY response inhibition test”; Garavan et al., 1999; Garavan, 2002; Roche et al., 2005; Chuah et al., 2006; Bernardi et al., 2015). During this task, capital letters X and Y are presented in a serial, alternating order at a rate of one per second. Participants were instructed to press a button for each stimulus that followed a different one (Go) and to withhold their response when two identical stimuli followed each other (NoGo). Each Go/NoGo task run lasted 5 min and comprised 300 stimuli (for a total of 600 stimuli per test block), 10% of which represented “lures” requiring withholding.

During the training session, each participant was presented with five runs of the Go-NoGo task in which a decrement of 100 ms in the duration of stimulus presentation was applied at each following trial. In particular, the duration of the stimulus varied from 900 ms (and 100 ms of interstimulus interval) to 500 ms (and 500 ms of interstimulus interval). This procedure was performed to identify the stimulus duration that was associated with a rate of commission errors corresponding to ∼50% in each participant. This approach was applied to avoid potential ceiling or flooring effects in the number of commission errors (Garavan, 2002; Chuah et al., 2006).

### Behavioral tasks

The HCD condition included computerized tasks based on impulse control, decision-making, and conflict resolution that were selected to engage as much as possible the so-called executive functions and their related brain networks. The LCD condition included a modified version of the same tasks employed in the HCD condition, adjusted to require no or minimal exertion of self-control.

#### Emotion suppression task

In the emotion suppression task (Baumeister et al., 1998; Dang, 2018), participants watched a series of brief video clips showing humans and/or animals in amusing situations. Participants were explicitly requested to completely suppress their facial reactions (e.g., smiling or laughing) while performing the HCD condition, whereas they were left free to express their emotional responses during the LCD session. Compliance with the task was assessed using a camera pointing at the participant's face (Avvenuti et al., 2021).

#### False response task

This task represents a modified version of the response conflict task adopted in previous work (Bernardi et al., 2015). Subjects were presented in random order with 180 simple questions (e.g., “How many fingers in one hand?”) and two possible answers, one false (e.g., “10”) and one correct (e.g., “5”). Participants were instructed to give, as fast and as accurately as possible, either the correct or wrong response according to a green/red sign which was presented below each question. The time limit for providing an answer was set to 2,000 ms. During the HCD condition, the sign's color was randomly assigned for each stimulus, while it remained always green in the LCD session.

#### Stroop task

The Stroop task is a widely known psychological test that requires selective attention, processing speed, and the ability to inhibit an automatic response (Stroop, 1935; Dang, 2018). This task included two repetitions of two distinct runs for a total of four runs. The stimuli consisted of four color words (i.e., “RED,” “YELLOW,” “GREEN,” “BLUE”) presented in red, yellow, green, or blue ink. In two runs, participants were instructed to indicate the color name represented by the word (i.e., ignoring the ink color), while in the other two runs, they had to indicate the ink color (i.e., ignoring the color name). In the HCD session, the color name and the ink color could be either congruent (i.e., color ink and color name were matched) or incongruent (i.e., the color ink and the color name did not match), whereas in the LCD condition stimuli were always congruent (i.e., color ink and color name were always matched).

### Performance evaluation and statistical analyses

Due to the relatively small number of NoGo trials compared with Go trials, we decided to aggregate the data obtained from blocks T1 and T2, naming these aggregated blocks as “post-HCD/LCD.” This was done to estimate more reliably the ERPs (see below) on both correct withholds and commission errors in NoGo trials. The change in performance level post-HCD/LCD was measured as the difference in percentage of commission errors on NoGo trials (%CE) and as the percentage of hits on Go trials (%HIT) relative to the baseline block. We also measured the difference in reaction time (RT) pre- and post-HCD/LCD. A paired-sample nonparametric test (Wilcoxon signed-rank test) was employed to assess variations in these measures compared with the baseline block. Similar tests were used to assess possible differences between HCD and LCD sessions in relative baseline-to-post-block variations. Effect sizes were calculated using rank-biserial correlation (RBC). Statistics are reported as mean ± standard deviation unless otherwise specified.

Since one of the observed effects of the HCD condition was the reduction in reaction times and the appearance in some subjects of a bimodal distribution including a Fast Trials (FT) peak in addition to the Standard Trial (ST) peak (see Results), we decided to conduct a more detailed analysis to characterize this phenomenon. For each subject, the probability density function (pdf) was estimated for all reaction times in each block and session (both hits and commission errors), using kernel density estimation. A Gaussian function was used as the kernel and the Improved Sheather–Jones algorithm was employed for bandwidth selection. For each subject, we calculated the difference between the pdf post-HCD and post-LCD with their respective baseline and we used the last point between 100 and 250 ms where this change in sign occurred to identify the separation line between FTs and STs. We used the median of all inversion points found as the cutoff for all subjects.

### EEG data analysis

EEG processing and analysis were conducted using EEGLAB (Delorme and Makeig, 2004), along with custom scripts in Matlab and Python. Continuous EEG recordings performed during each Go/NoGo run were band-pass filtered between 1 and 45 Hz using a finite impulse response (FIR) filter and rereferenced to the average reference. Bad channels were automatically removed by calculating their kurtosis values and excluding those with an absolute *z*-score higher than 5. Removed channels were interpolated with a spherical interpolation. Then, an independent component analysis (ICA) was performed, and the obtained components were automatically labeled using ICLabel (Pion-Tonachini et al., 2019). Components associated with artifacts such as eye movements, cardiac activity, and muscle activity were removed.

#### ERP analyses

Processed EEG data were epoched to generate both stimulus-locked and response-locked ERPs. Stimulus-locked epochs were selected within a window ranging from −100 to +1,000 ms, with the first 100 ms serving as the baseline. While stimulus-locked ERPs were computed for all types of trials [hits, correct withhold (CW) and commission errors (CE)], response-locked ERPs were calculated only for commission errors to analyze the error-related negativity (ERN). This analysis was performed within a window ranging from −300 to +500 ms, using the range −300 to −200 ms as baseline. For each subject and trial type, we computed the average signal across all epochs. The ERPs of each subject were visually inspected to verify the presence of distinguishable N200, ERN, and P300 components. Subjects who did not exhibit discernible ERP responses in specific trials or conditions were excluded from related analyses. Out of the starting set of 26 subjects, one subject was excluded from all analyses due to poor-quality EEG data. The final dataset is hence composed of *n* = 25 subjects for all the analyses unless otherwise stated. The analysis on commission errors epochs of the LCD sessions was performed only on *n* = 23 subjects, as two subjects did not show clearly distinguishable ERP components.

Different ERP components were analyzed based on trial type, using the area under the curve (AUC) around the corresponding peak of interest. The peak for each component was determined by searching within a component-specific window for both stimulus-locked [anterior P200: (140, 250) ms, posterior P200: (160, 280) ms, N200: (200, 350) ms, P300: (250, 600) ms] and response-locked epochs [ERN: (−50, 150), P300: (100, 400) ms] all possible instants *i* where the corresponding potential *p* satisfied the condition:

$$p_{i-1}\left\langle \;p_{i}\;\mathrm{and}\;p_{i}\right\rangle \;p_{i+1}\;\;(\mathrm{inverted}\;\mathrm{operators}\;\mathrm{for}\;\mathrm{negative}\;\mathrm{peaks}),$$

Among all possible identified peaks, we selected the one having the highest absolute potential. Once the peak was identified, the AUC was calculated within a time window of 60 ms centered around it, employing the trapezoidal method. The AUC variation between baseline and post-task blocks, as well as differences between sessions, were assessed using Wilcoxon signed-rank tests.

#### FT analysis

To analyze the functional underpinnings of FTs (see Results), we computed the response-locked ERPs of FTs and STs in hits trials in a time window of −500 to +500 ms. Baseline correction for both FTs and STs was applied using the signal from −100 ms to stimulus onset (Kelly and O’Connell, 2013). To identify the scalp regions exhibiting differences in FTs and STs before the response, we calculated the average potential between −150 and 0 ms for each electrode. Then, a paired-sample statistical test (Wilcoxon signed-rank test, FDR correction Extended Data Fig. 3-1) was applied, and specific regions of interest (anterior and posterior ROIs) were chosen for further analyses (see Results). The response-locked ERPs from electrodes within the two ROIs were averaged to obtain ROI-specific ERPs. Potential differences between STs and FTs were investigated using time-point-wise Wilcoxon signed-rank tests and an FDR correction for multiple comparisons. In addition, we marked as significant only clusters of contiguous significant time-points lasting at least 30 ms. To quantify the build-up activity observed in the posterior ROI, we extracted for each subject the EEG activity from stimulus onset to ERP peak (detected using the same method employed for detecting the main ERP components). The slope magnitude of the signal deflection was quantified using a linear mixed-effect model.

We analyzed also signal power differences in a window of 300 ms before the stimulus onset. For this, we computed an estimate of the power spectral density in each channel, employing the modified periodogram method with a Hamming window of the same length as the epoch. We then calculated the average power for each subject in three bands of interest: theta (4–7 Hz), alpha (8–13 Hz), and beta (13–30 Hz). Differences between FTs and STs for each band and each electrode were assessed using a Wilcoxon signed-rank test with FDR correction. Given the brief duration of the window, differences in the delta band were not examined. We repeated this analysis also in the response-locked epochs.

## Results

**Figure 1.**
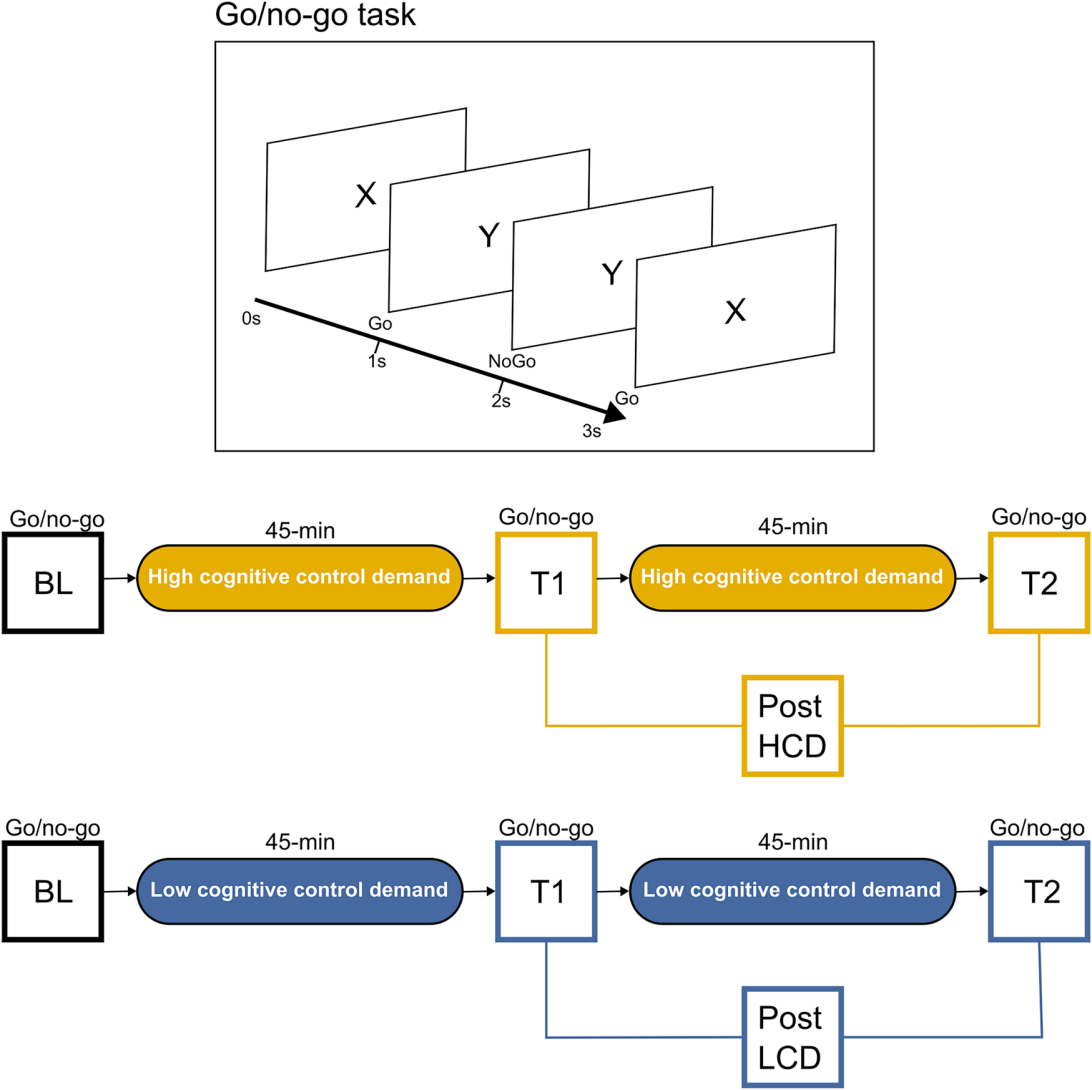
Experimental design. Participants completed two experimental sessions, one requiring the completion of two task blocks involving either cognitively demanding tasks (HCD) or a modified version of the same tasks requiring no minimal cognitive effort (LCD). A Go/NoGo task was completed at baseline (BL) and after each task block (T1, T2).

To investigate the effects of extended cognitive load on impulse control and its neural underpinnings, we asked 25 subjects to perform a Go/NoGo task after rest and then again after two 45-min-long task practice sessions including either high cognitive demand (HCD) or low cognitive demand (LCD) tasks (Fig. 1; Materials and Methods). We recorded and compared behavioral performance and hd-EEG during the Go/NoGo tasks before and after the LCD and HCD tasks.

We first assessed the effects of HCD and LCD tasks on self-reported alertness, sleepiness, perceived effort, mood, and motivation. We found that alertness, mood, and motivation decreased from baseline to post-task period, while sleepiness and perceived effort increased (Table 1) with no significant differences between LCD and HCD.

**Table 1.** Self-reported condition following high cognitive demand (HCD) and low cognitive demand (LCD) sessions

	ΔHCD	ΔLCD	ΔHCD vs ΔLCD
Alertness	1.48 ± 1.52 (RBC = −0.81, *W* = 29.5, *n* = 25, *p* = 0.0005*)	−1.82 ± 1.24 (RBC = −1, *W* = 0, *n* = 23, *p* = 0.0001*)	0.34 ± 1.40 (RBC = 0.24, *W* = 71.5, *n* = 19, *p* > 0.1
Sleepiness	1.50 ± 1.46 (RBC = 0.89, *W* = 16, *n* = 24, *p* = 0.0005*)	1.96 ± 1.50 (RBC = 0.94, *W* = 9, *n* = 24, *p* = 0.0003*)	−0.46 ± 1.14 (RBC = −0.44, *W* = 83, *n* = 24, *p* > 0.1)
Effort	2.76 ± 1.80 (RBC = 0.98, *W* = 2.5, *n* = 24, *p* = 0.0001*)	2.38 ± 1.88 (RBC = 0.98, *W* = 2, *n* = 24, *p* = 0.0001*)	0.38 ± 2.07 (RBC = 0.39, *W* = 83.5, *n* = 23, *p* > 0.1)
Mood	−0.36 ± 0.68 (RBC = −0.71, *W* = 15, *n* = 14, *p* = 0.075)	−0.66 ± 0.70 (RBC = −0.89, *W* = 8, *n* = 17, *p* = 0.005*)	0.30 ± 0.62 (RBC = 0.62, *W* = 25.5, *n* = 16, *p* > 0.1)
Motivation	−0.52 ± 0.80 (RBC = −0.84, *W* = 8, *n* = 14, *p* = 0.02*)	−0.64 ± 0.67 (RBC = −0.94, *W* = 5, *n* = 18, *p* = 0.0015*)	0.12 ± 1.82 (RBC = 0.16, *W* = 44, *n* = 16, *p* > 0.1)

The second and third columns show the variation (mean ± SD) relative to the baseline of the Likert scales for alertness, sleepiness, effort, mood, and motivation after the two types of session. In the fourth column is reported the differences between post-HCD and post-LCD variations (mean ± SD). Each column reports effect size, statistic, number of nonzero differences, and *p* values of Wilcoxon test post-Bonferroni correction (*p < 0.05).

### Effects of cognitive fatigue on behavioral performance

**Figure 2.**
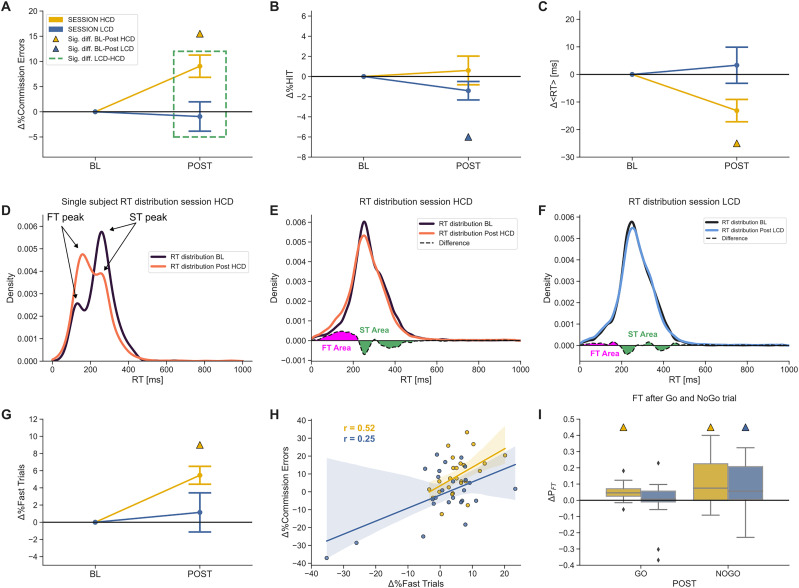
Performance in Go/NoGo tasks following High Cognitive Demand (HCD) and Low Cognitive Demand (LCD) sessions. ***A***, Relative changes in the percentage of commission errors between Go/NoGo blocks performed before (baseline; BL) and after (POST) practice with HCD (yellow) or LCD (blue) tasks (mean ± SEM). Here and in panels ***B***, ***C***, ***G***, and ***H***, yellow (blue) triangles indicate significant differences between POST and BL (*p* < 0.05, Wilcoxon test) for HCD (LCD), and dashed boxes indicate significant differences between HCD and LCD. ***B***, Same as ***A*** for the relative variation in the percentage of hits (%HIT). ***C***, Same as ***A*** for the relative variation in reaction time in hits (RT). ***D***, Distribution of RTs for a representative subject (BL in black and POST in orange) during the HCD session. ***E***, RT distribution for all subjects in HCD sessions. The magenta and green areas indicate the positive (Fast Trial area, FT) and negative (Standard Trial area, ST) differences between the two distributions (POST-BL), respectively. ***F***, same as ***E*** for LCD. ***G***, Same as ***A*** for the percentage of fast trials. ***H***, Correlation between relative changes in the percentage of fast trials and relative changes in the percentage of commission errors (POST-BL; HCD, yellow; LCD, blue). ***I***, Relative change in the probability of occurrence of an FT between baseline and post-HCD/LCD after Go or NoGo trials. See Extended Data Figure 2-1 for the overall difference between post-Go and post-NoGo FT occurrence probability.

We investigated to which extent practice with HCD or LCD tasks affected subsequent impulse control as measured through the Go/NoGo task. The fraction of commission errors increased significantly in the HCD condition (BL-HCD %CE = 39.03%, post-HCD %CE: 48.08%; Δ%CE = 9.05% ± 11.03%, RBC = 0.76, *W* = 35, *n* = 24, *p* = 0.001; Wilcoxon test) but not in the LCD condition (Fig. 2*A*; BL-LCD %CE = 44.03%, post-LCD %CE: 43.08%; Δ%CE = −0.94% ± 14.56%, RBC = 0.02, *W* = 159, *n* = 25, *p* = 0.93; Wilcoxon test). The relative variation in the number of commission errors was significantly different between HCD and LCD (Fig. 2*A*; RBC = 0.59, *W* = 65.5, *n* = 25, *p* = 0.008; Wilcoxon test). The fraction of hits decreased significantly in the LCD (BL-LCD %HIT = 97.20%, post-LCD %HIT: 95.79%; Δ%HIT = −1.41% ± 4.58%, RBC = −0.59, *W* = 61, *n* = 24, *p* = 0.0108; Wilcoxon test) but not in the HCD (BL-HCD %HIT = 96.64%, post-HCD %HIT: 97.25%; Δ%HIT = 0.6% ± 7.11%, RBC = −0.33, *W* = 83.5, *n* = 22, *p* = 0.16; Wilcoxon test) condition (Fig. 2*B*).

10.1523/ENEURO.0354-24.2024.f2-1 Figure 2-1 Probability of FT occurrence after a Go or NoGo trial calculated on all trials for each subject (p < 0.00001, Wilcoxon Test). Download Figure 2-1, TIF file.

The mean reaction time (RT) in hits decreased in HCD (BL-HCD RT = 279.43 ms, post-HCD RT: 266.30 ms; ΔRT = −13.13 ms ± 20.26 ms, RBC = −0.61, *W* = 62, *n* = 25, *p* = 0.0055; Wilcoxon test) but not in LCD (Fig. 2*C*; BL-LCD RT = 273.03 ms, post-LCD RT: 276.37 ms; ΔRT = 3.33 ms ± 32.80 ms, RBC = −0.04, *W* = 156, *n* = 25, *p* = 0.87; Wilcoxon test), and the relative variation did not significantly differ between conditions by a small margin (RBC = −0.42, *W* = 93, *n* = 25, *p* = 0.06; Wilcoxon test). Thus, the HCD condition was associated with faster responses and an increase in the percentage of commission errors, while the LCD condition was associated with a decrease in hits.

A finer analysis of RT distributions in the HCD condition revealed that the relative post-HCD decrease in RT was explained by an increase in the number of fast responses (i.e., responses for which RT < 200 ms; see Fig. 2*D* for a representative subject). For each subject, we identified the lowest RT at which the difference between the RT probability density function postsession and at baseline (see Materials and Methods) changed in sign (Fig. 2*E*,*F*). This inversion point corresponded to RT = 203 ± 59 ms (median ± IQR). Based on this observation, we classified trials with RT < 200 ms as Fast Trials (FTs) and trials with RT > 200 ms as Standard Trials (STs).

We next assessed baseline to post-task variations in the percentage of FTs (Fig. 2*G*). We found a significant %FT increase in the HCD condition (BL-HCD %FT = 14.31%, post-HCD %FT: 19.78%; Δ%FT: 5.47% ± 5.20%, RBC = 0.90, *W* = 15, *n* = 25, *p* = 0.000008, Wilcoxon test), while no significant changes were observed in the LCD condition (BL-LCD %FT = 15.34%, post-LCD %FT: 16.49%; Δ%FT: 1.15% ± 11.39%, RBC = 0.37, *W* = 101, *n* = 25, *p* = 0.101; Wilcoxon test). The post-HCD increase in FTs was sufficient to explain the overall decrease in RTs as this effect was no longer present when FTs were removed (BL-HCD RT: 306.59 ms, post-HCD RT: 300.78 ms, ΔRT = −5.81 ms ± 28.96 ms, RBC = −0.15, *W* = 137, *n* = 25, *p* = 0.50; Wilcoxon test). Furthermore, the difference between the two sessions in %CE was no longer significant after the removal of FT (HCD Δ%CE: 6.23% ± 10.20%, LCD Δ%CE: 0.63% ± 11.43%; RBC = 0.41, *W* = 94.5, *n* = 25, *p* = 0.07; Wilcoxon test). Of note, the baseline versus post-task variation in the percentage of FTs in HCD was significantly correlated across subjects with the increase in commission errors (*r* = 0.52, *p* = 0.008; Spearman's coefficient), while this did not occur after LCD tasks (*r* = 0.25, *p* = 0.23; Spearman's coefficient; Fig. 2*H*).

Finally, we examined whether the FTs occurrence was affected by previous responses. Overall, FT occurred more often after NoGo trials (post Go %FT 0.14 ± 0.10, post-NoGo %FT 0.44 ± 0.19, RBC = 1, *W* = 0, *n* = 25, *p* = 5.96 × 10^−8^; Wilcoxon test; Extended Data Fig. 2-1). The probability of FT after NoGo trials increased relatively to baseline after both HCD (post NoGo BL %FT 37.84%, post NoGo post-HCD %FT 49.47%; Δpost-NoGo 11.63% ± 15.10%, RBC = 0.70, *W* = 44, *n* = 24, *p* = 0.002; Wilcoxon test) and LCD (post NoGo BL %FT 36.18%, post NoGo post-LCD %FT: 45.15%; Δpost-NoGo 8.98% ± 11.54%, RBC = 0.62 *W* = 62, *n* = 25, *p* = 0.005; Wilcoxon test). The probability of FT occurrence following Go trials increased significantly relative to baseline only after the HCD session (post Go BL %FT 11.43%, post Go post-HCD %FT 16.25%; Δpost-Go 4.81% ± 4.78%, RBC = 0.85, *W* = 24, *n* = 25, *p* = 4.54 × 10^−5^; Wilcoxon test), and not after the LCD session (post Go BL %FT 12.77%, post Go post-LCD %FT 13.03%; Δpost-Go 0.26% ± 11.54%, RBC = 0.29, *W* = 115.0, *n* = 25, *p* = 0.20; Wilcoxon test). However, there was no interaction of session and trial type in determining the relative changes (two-way rmANOVA on rankings, *p* > 0.1).

### Cortical activity underlying fast trials

Given the functional relevance of FT described in the previous section, we compared the cortical activity underlying FTs and STs.

**Figure 3.**
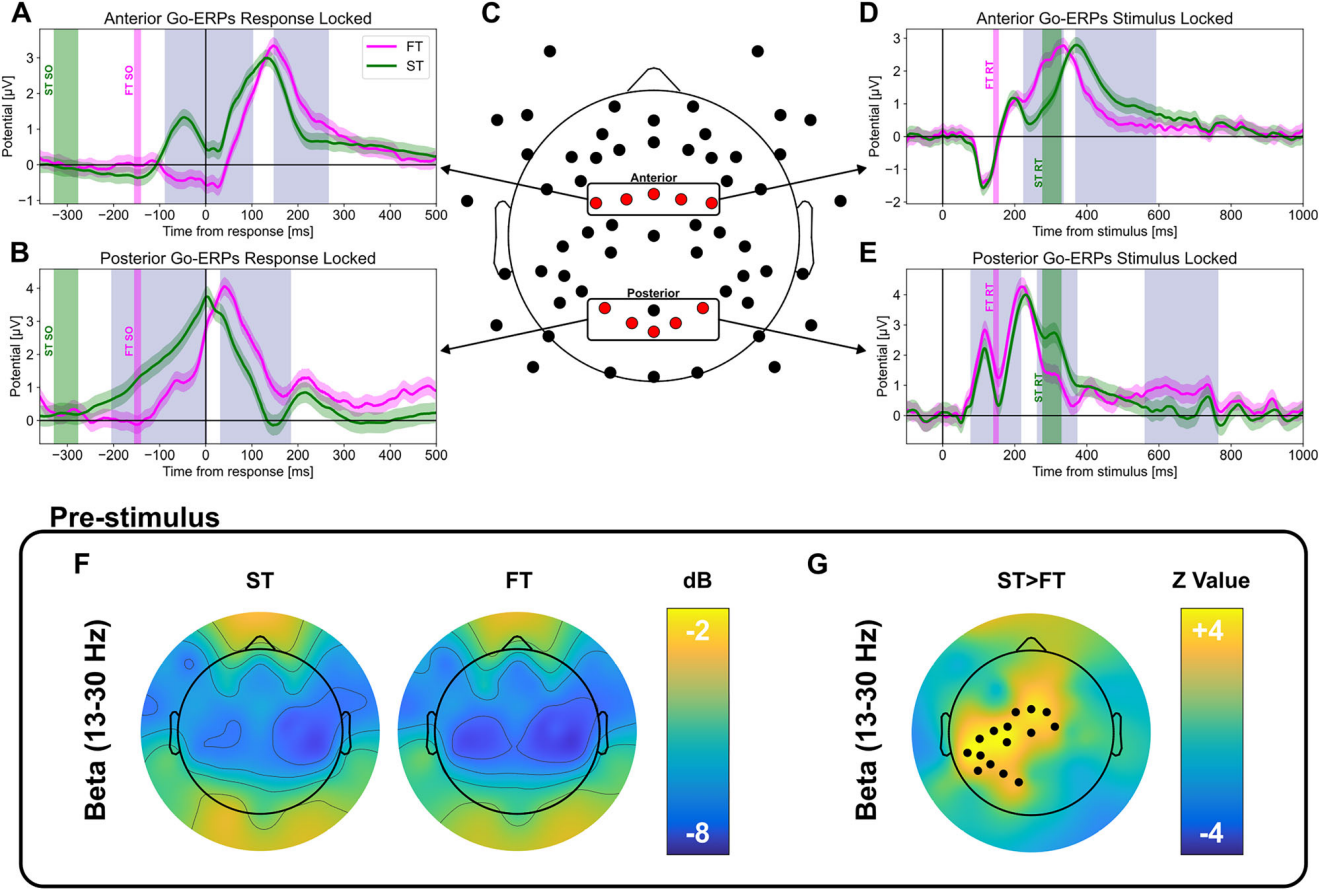
Event-related potentials (ERPs) in cortical activity associated with standard trials (ST) and fast trials (FT). ***A***, Response-locked ERPs in the anterior ROI for FTs and STs for correct Go trials. The blue area indicates the time interval in which a significant difference between FTs and STs was observed (*p* < 0.05, FDR correction, Wilcoxon test). The green and magenta vertical bands show the interquartile range of stimulus onset for STs and FTs, respectively. ***B***, Same as ***A*** but for the posterior ROI. ***C***, Electrode montage. Electrodes belonging to the anterior and posterior regions of interest (ROIs) are colored in red (see Materials and Methods and Extended Data Fig. 3-1). ***D***, Same as ***A*** but for stimulus-locked ERPs. In this case, the green and magenta vertical band indicates the interquartile range of reaction time for STs and FTs, respectively. ***E***, Same as ***D*** but for the posterior ROI. Extended Data Figures 3-2–3-5 show the difference in ERPs for FTs between sessions respectively in the ROIs and epochs for ***A***, ***B***, ***D***, and ***E***. ***F***, Topographic distribution of beta power (13–30 Hz) for STs (left) and FTs (right) prestimulus epochs (−300, 0) ms. ***G***, Topographic difference between ST and FTs for the beta band. Black dots represent the electrodes for which a significant difference has been found (*p* < 0.05 FDR correction; Wilcoxon test). Extended Data Figure 3-6 shows the same analyses displayed in ***F*** and ***G*** for the theta and alpha bands. See Extended Data Figure 3-7 for the topographic distribution difference between FTs and STs in the response-locked epochs for theta, alpha, and beta power bands.

We first analyzed the EEG ERPs time aligned to correct hits in Go trials. Of note, the experimental condition (HCD, LCD) did not affect the shape of associated ERPs (Extended Data Figs. 3-2, 3-3), so we averaged data over HCD and LCD recordings. In frontal electrodes (anterior ROI; Fig. 3*C*), the preresponse interval was associated with stronger activity in STs than in FTs (Fig. 3*A*; *p* < 0.05 Wilcoxon test, FDR correction). Indeed, STs displayed a clear ERP peak occurring −46 ms ± 18 ms (median ± IQR) before the behavioral response, while no peak was present in FTs. Postresponse, both FTs and STs displayed a peak. In parietal electrodes (posterior ROI; Fig. 3*B*), we observed a ramp-up of the activity that started after stimulus presentation and peaked at response time for STs (latency, 4 ms ± 18 ms) but significantly later (RBC = 0.83, *W* = 25.5, *n* = 24, *p* = 0.0003; Wilcoxon test) for FTs (latency, 40 ms ± 18 ms). However, given the shorter reaction time in FTs, the ERP response rose faster for FTs (slope, 21.18 μV/s) than that for STs (slope, 12.41 μV/s; Fig. 3*B*). Overall, FTs were characterized by a lesser activity preceding the response, in particular in the anterior region.

10.1523/ENEURO.0354-24.2024.f3-1 Figure 3-1 Comparison of the potential distribution on the scalp between FTs and STs in the 150  ms preceding the response. Black dots represent the electrodes for which a significant difference has been found (Wilcoxon test, FDR correction). Download Figure 3-1, TIF file.

10.1523/ENEURO.0354-24.2024.f3-2 Figure 3-2 Between sessions differences in anterior ROI for FTs response-locked epochs. The differences are assessed in the same way in Figure 3A, B, D, E. Download Figure 3-2, TIF file.

10.1523/ENEURO.0354-24.2024.f3-3 Figure 3-3 Between sessions differences in posterior ROI for FTs response-locked epochs. The differences are assessed in the same way in Figure 3A, B, D, E. Download Figure 3-3, TIF file.

**Figure 4.**
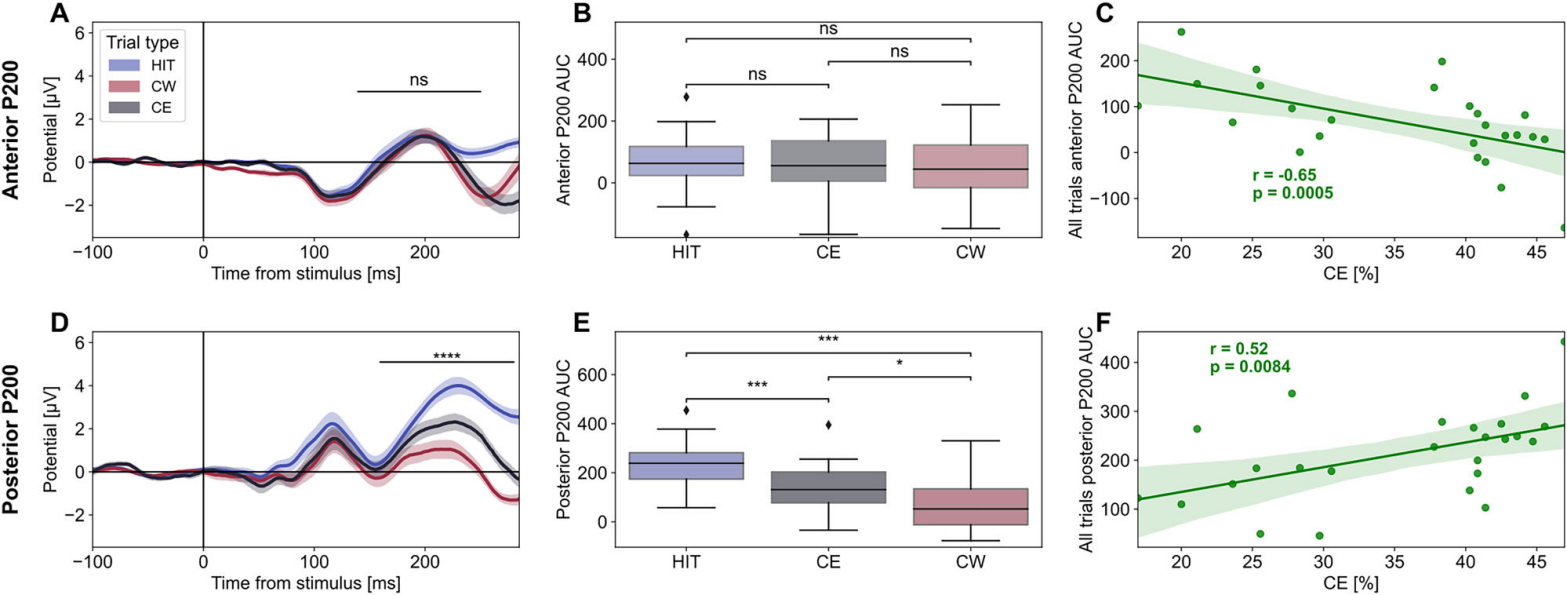
Anterior and posterior P200 in STs role in action selection and cognitive control. ***A***, ***D***, Stimulus-locked ERPs of hits (HIT, blue), correct withholds (CW, red), and commission errors (CE, black) in STs, as calculated from all sessions and conditions in the anterior (***A***) and posterior (***D***) ROI. The black horizontal line above the anterior and posterior P200 indicates the area where peaks were searched for (ns: *p* > 0.05, ****: *p* < 0.0001; Friedman test). ***B***, ***E***, Areas under the curve (AUC) divided by trial type in the anterior (***B***) and posterior (***E***) ROI (ns: *p* > 0.05, *: *p* < 0.05, **: *p* < 0.01, ***: *p* < 0.001; Nemenyi test). ***C***, Correlation between the percentage of commission errors and anterior P200 AUC, as calculated using all trial types in the anterior ROI. ***F***, Same as ***C*** but for the posterior ROI.

10.1523/ENEURO.0354-24.2024.f3-4 Figure 3-4 Between sessions differences in anterior ROI for FTs stimulus-locked epochs. The differences are assessed in the same way in Figure 3A, B, D, E. Download Figure 3-4, TIF file.

**Figure 5.**
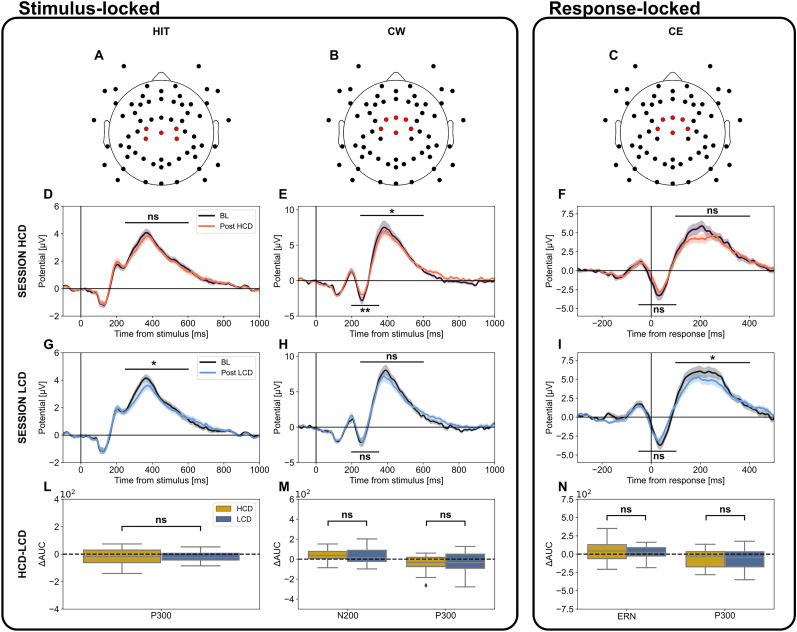
Stimulus-locked ERPs during HIT, CW, and CE trials following High Cognitive Demand (HCD) and Low Cognitive Demand (LCD) sessions. ***A*–*C***, Electrode montage. Electrodes in red were used to compute the ERPs displayed below in each topographic plot. ***A***, For hit trials; ***B***, correct withholds (CW); and ***C***, commission errors (CE). ***D***, ***E***, ***G***, ***H***, Average stimulus-locked ERPs for hits (***D***, ***G***) and CW (***E***, ***H***) at baseline and after HCD (***D***, ***E***) or LCD (***G***, ***H***) tasks. ***F***, ***I***, Average response-locked ERPs for commission errors at baseline and post-HCD (***F***) or LCD (***I***) tasks. The black horizontal line above (P300) or below (N200, ERN) the ERP components indicates the area where peaks were searched for (ns: *p* < 0.05, *: *p* < 0.05, **: *p* < 0.01; Wilcoxon test). ***L***, Comparison between post-HCD (yellow) and post-LCD (blue) variations from baseline for the P300 AUC in hit trials. ***M***, Same as (***L***) for CW trials and both N200 and P300 components. ***N***, same as (***L***) for CE trials and both ERN and P300 components (ns: *p* > 0.05, *: *p* < 0.05; Wilcoxon test).

10.1523/ENEURO.0354-24.2024.f3-5 Figure 3-5 Between sessions differences in posterior ROI for FTs stimulus-locked epochs. The differences are assessed in the same way in Figure 3A, B, D, E. Download Figure 3-5, TIF file.

10.1523/ENEURO.0354-24.2024.f3-6 Figure 3-6 Theta and Alpha band FT-ST pre-stimulus [-300,0]. Black dots represent the electrodes for which a significant difference has been found as in Figure 3 F, G (p < 0.05, Wilcoxon test, FDR correction). Download Figure 3-6, TIF file.

10.1523/ENEURO.0354-24.2024.f3-7 Figure 3-7 Theta, alpha, and Beta band power difference between ST and FT in response-locked. Black dots represent the electrodes for which a significant difference has been found as in Figure 3 F, G (p < 0.05,Wilcoxon test, FDR correction). Download Figure 3-7, TIF file.

We next examined the differences between FTs and STs in stimulus-locked ERPs in the same ROIs. Again, the experimental condition (HCD, LCD) did not affect the shape of associated ERPs (Extended Data Figs. 3-4–3-5), so we averaged data over HCD and LCD recordings. In frontal electrodes (Fig. 3*D*), we observed during STs a standard P200 neural response and a later P300 neural potential following the behavioral response, while the P200 for FTs occurred after the response and overlapped with the P300 potential. In the posterior electrodes (Fig. 3*E*), both FTs and STs showed increased activity ∼100 ms after stimulus onset, preceding the behavioral response, with stronger activity in FTs (Fig. 3*E*; *p* < 0.05, Wilcoxon test, FDR correction). Both FTs and STs displayed also a second peak of activity ∼250 ms after the stimulus onset—but note that this peak preceded behavioral response in FTs while it followed behavioral response in STs. Overall, the poststimulus processing is similar in FTs and STs but in the former case the response occurs before it is completed.

We hypothesized then that FTs and STs could originate due to different activities preceding stimulus onset. The spectral analysis performed in a 300 ms prestimulus window revealed a significant higher beta power (13–30 Hz) in STs compared with FTs in the centroparietal area contralateral to the hand that executed the response (Fig. 3*G*; *p* < 0.05, Wilcoxon test, FDR correction). No differences were found instead between FTs and STs in the theta (4–7 Hz) and alpha (8–13 Hz) bands (Extended Data Fig. 3-6). Instead, in the response-locked spectrum, we observed a significantly lower theta power on frontal region and higher alpha power on occipital region in FTs compared with STs. No difference was found in the beta band (Extended Data Fig. 3-7; *p* < 0.05, Wilcoxon test, FDR correction).

### Cortical activity underlying commission errors in standard trials

Commission errors are correlated with the fraction of FTs, but on average only 19% of CEs (first and third quartiles across subjects: [6.87, 27.48] %) were performed during FTs, and the remaining were performed during STs. We investigated then whether commission errors were associated with specific features of the stimulus-locked ERP response in standard trials. We analyzed the P200 components identified in the anterior ROI and posterior ROI during STs putting together HCD and LCD (Fig. 3*D*,*E*). We measured the area under the curve (AUC) of the peaks of both the anterior (Fig. 4*A*) and the posterior P200 (Fig. 4*D*) recorded during the three types of responses: hits, correct withholds, and commission errors. In the anterior region, there were no P200 differences across responses (Fig. 4*B*; Kendall's *W* = 0.02, *Q* = 1.28, *p* = 0.52, Friedman test), but across subjects P200 AUC significantly anticorrelated with the percentage of commission errors across all sessions and blocks (*r* = −0.65, *p* = 0.0005, Spearman's coefficient; Fig. 4*C*). In the posterior region, we observed a significant difference in P200 across all three types of trials (Kendall's *W* = 0.82, *Q* = 41.04, *p* = 1.25 × 10^−9^, Friedman test; Fig. 4*E*). In particular, the smallest P200 AUC was found for CW trials, while the largest was observed for HIT trials. In this case, a positive intersubject correlation between the posterior P200 AUC and the percentage of commission errors was found (*r* = 0.52, *p* = 0.0084, Spearman's coefficient; Fig. 4*F*). Furthermore, a negative intersubject correlation was observed between the posterior P200 AUC calculated on hits trials and the average reaction time computed on the same trial type across all sessions and blocks (*r* = −0.57, *p* = 0.0027, Spearman's coefficient).

These results suggest that commission errors during standard trials might originate from posterior P200 significantly larger than the ones usually associated with withdrawal and from a lower anterior P200.

### Frontocentral event-related potentials in standard trial modulation by HCD and LCD

In the previous sections, we have observed how the relative occurrence of FT and STs changed differentially after HCD or LCD, while anterior and posterior ERPs of each trial type did not change depending on the previous cognitive load. However, according to previous studies (Polich, 2007; Stock et al., 2016; Yin et al., 2016), the P300 is localized close to central electrodes (around Cz; Fig. 5*A*), while both N200 and ERN, as well as the P300 of correct withhold and commission error trials, exhibit a frontocentral distribution (Fig. 5*B*,*C*; Nieuwenhuis et al., 2003; Donkers and Van Boxtel, 2004; Polich, 2007; Kato et al., 2009; Huster et al., 2013; Iannaccone et al., 2015; Wang et al., 2020; Kirschner et al., 2021). We investigated then if these components changed between HCD and LCD.

The hit-related P300 component did not change significantly after HCD tasks (Fig. 5*D*; RBC = −0.23, *W* = 125, *n* = 25, *p* = 0.32, Wilcoxon test), while its amplitude decreased significantly after LCD tasks (Fig. 5*G*; RBC = −0.46, *W* = 87, *n* = 25 *p* = 0.042, Wilcoxon test). However, no significant differences were found between relative changes after HCD and LCD tasks (Fig. 5*L*; RBC = 0.15, *W* = 137, *n* = 25, *p* = 0.50, Wilcoxon test).

A significant reduction of P300 was observed in correct withhold trials after practice with HCD (RBC = −0.5, *W* = 82, *n* = 25, *p* = 0.029) but not with LCD (RBC = −0.3, *W* = 114, *n* = 25, *p* = 0.20) tasks (Fig. 5*E*). We found a significant amplitude decrease for the N200 component after practice with HCD (RBC = 0.60, *W* = 65, *n* = 25, *p* = 0.007) but not LCD (RBC = 0.32, *W* = 111, *n* = 25, *p* = 0.17) tasks (Fig. 5*E*). Again, however, no significant differences emerged between HCD and LCD experimental conditions (Fig. 5*M*; N200: RBC = 0.21, *W* = 127, *n* = 25. *p* = 0.35, P300: RBC = 0.009, *W* = 161, *n* = 25, *p* = 0.97, Wilcoxon test).

Finally, we examined the effect of extended task practice on the ERPs of commission errors. No significant ERN changes were found after both HCD (Fig. 5*F*; RBC = 0.29, *W* = 115, *n* = 25, *p* = 0.20, Wilcoxon test) and LCD tasks (Fig. 5*I*; RBC = 0.35, *W* = 90, *n* = 25, *p* = 0.15, Wilcoxon test). For the P300 component, we observed a significant reduction for the LCD (Fig. 5*I*; RBC = −0.5, *W* = 69, *n* = 23, *p* = 0.035, Wilcoxon test) but not for the HCD session (Fig. 5*F*; RBC = −0.35, *W* = 106, *n* = 25, *p* = 0.13, Wilcoxon test). No significant differences were found between HCD and LCD conditions for both ERN and P300 (Fig. 5*M*; ERN: RBC = 0.12, *W* = 121, *n* = 23, *p* = 0.62, P3: RBC = −0.028, *W* = 134, *n*  = 23, *p* = 0.91, Wilcoxon test).

Overall, when removing FTs, differences between EEG recordings following HCD and LCD are negligible. This suggests that behavioral differences could be largely explained by the dynamics underlying FTs.

## Discussion

We found that extended practice with high cognitive demand tasks involving response inhibition was associated with decreased reaction time and increased commission errors. Commission errors following HCD sessions were in turn associated with the appearance of fast, automatic neural responses characterized by distinctive ERP shapes. Interestingly, when FTs were removed, we observed no significant differences between the ERP components following HCD and LCD sessions in both Go and NoGo trials. This suggests that behavioral changes following experience-dependent cognitive fatigue mainly depend on the more frequent occurrence of the fast neural response modality.

### Behavioral effects of extended cognitive load

Previous work showed that cognitive fatigue induced by extended task practice is associated with a deterioration of response inhibition performance (Kato et al., 2009; Möckel et al., 2015; Guo et al., 2018). The specific effects may differ in part depending on the task used to measure behavioral performance and include increases in commission errors with or without a decrease in reaction time and/or increases in reaction time and the number of missing responses. These studies also commonly reported an increased behavioral instability, with strong fluctuations in response accuracy and reaction time.

Here we showed that extended practice with tasks requiring impulse control led to increased commission errors and decreased reaction time during a fast-paced Go/NoGo task. The occurrence of commission errors appeared to be related to an increased occurrence of fast, automatic responses in addition to standard response modalities. This might be due to the fact that fast automatic responses could be less energy demanding while preserving a good performance due to the prevalence of Go trials. In both HCD and LCD subjects, indeed, fast trials are more likely to occur after NoGo trials when a Go trial is expected. In HCD, mental fatigue increased the use of the less demanding but hastier strategy of fast automatic responses, leading to more commission errors. This effect was not significant after practice with identical tasks modified to remove the impulse control component. In this case, we observed tendencies toward an increased number of misses. Therefore, behavioral changes in the two experimental conditions appeared to point in almost opposite directions, with faster, automatic responses on the one hand (HCD) and more sluggish responses on the other hand (LCD). The observed differences might be better explained by the involvement of distinct functional mechanisms rather than by a graded involvement of the same mechanism as a function of cognitive demands. Indeed, relative behavioral changes observed in the LCD condition could reflect a tendency of participants to reduce focus and attention allocation when tasks are more monotonous and less stimulating (Balkin and Wesensten, 2011). Instead, behavioral changes observed in the HCD condition are consistent with use- and task-dependent cognitive fatigue and behavioral instability in which responses appeared to variably oscillate more often between a “standard modality” and a distinctive, fast, automatic modality.

### Neural changes induced by extended cognitive load

We next used EEG and ERP analysis to investigate the electrophysiological correlates of the different behavioral response modalities. We found that fast trials were characterized by distinctive electrophysiological correlates relative to standard trials. In particular, fast trials were preceded by less strong recruitment of left-lateralized, centroparietal brain areas relative to standard trials. According to previous work, higher power in the beta band contralateral to the hand used to produce behavioral responses could indicate a proactive response control (Tzagarakis et al., 2015; Muralidharan et al., 2019), which would be lacking or reduced for fast trials. In addition, we found that fast trials lacked a frontal ERP modulation in the ∼100 ms before action execution (anterior P200) that is instead present in standard trials. Furthermore, the fact that the posterior P200 reaches its peak after the response may indicate that the action was initiated before the evidence accumulation process was completed (O’Connell and Kelly, 2021).

To better characterize the differences between FT and ST, we performed an additional analysis on the two components of the ERPs that differed between the two types of responses (Fig. 4). The amplitude of the anterior P200 was not linked to the categorization of the stimulus type, as no differences were observed between Go and NoGo trials. However, subjects with a more pronounced component were more capable of exercising cognitive control and consequently made fewer commission errors. These results confirm that activation of the midfrontal cortex is crucial for applying proper cognitive control over actions (Simmonds et al., 2008; Forstmann et al., 2010; Cavanagh and Frank, 2014). On the other hand, the posterior P200 differed based on the type of trial. Specifically, the amplitude is greater when an action needs to be executed, while lower in the opposite case. The fact that in commission errors, the amplitude is a midpoint between Go and NoGo confirms that this component is linked to an evidence accumulation process. In this case, the same confidence as a Go trial was not achieved, but the accumulated evidence was sufficient to trigger the response. In addition, the positive correlation of the amplitude with the percentage of commission errors may indicate that a stronger posterior P200 is related to the presence of a bigger bias on the Go response. Overall, these findings indicate that fast trials may result from subjects’ minor tendency to complete the steps of the decision-making process preceding the response. In such instances, incoming sensory stimuli would trigger an automatic behavioral reaction, which may lead to errors if the stimulus is one required to withhold the response.

It is important to note that previous investigations found cognitive fatigue to be associated with a decrease in P300 and ERN amplitude (Lorist et al., 2005; Boksem et al., 2006; Kato et al., 2009; Möckel et al., 2015). These results have been suggested to reflect a reduced ability of the fatigued brain to allocate cognitive resources to the task and a compromised error-monitoring function (Kato et al., 2009).

In our present investigation, we observed a decrease in the amplitude of P300 for hits and commission error trials in the LCD session and both N200 and P300 during correct withholds in the HCD session. However, for none of the observed components, a difference in effect between the sessions was found. Therefore, our results indicate that ERP changes commonly observed in states of cognitive fatigue may not reflect functional alterations responsible for behavioral instability and commission errors. Instead, ERP changes may reflect more general variations induced by time-on-task potentially associated with global changes in alertness or motivation.

### Limitations

Analyses exploring the neural correlates of wrong (commission errors) and correct (correct withhold) NoGo trials were based on a relatively small number of trials. Indeed, the adopted task is based on the necessity to suppress a prepotent, automatic response induced by the rhythmic presentation of multiple Go trials. Therefore, the number of Go and NoGo trials was not balanced. However, we modulated task difficulty so that all participants had, at baseline, an error rate close to 50%. This allowed us to minimize the risk of possible ceiling or flooring effects and thus obtain trials corresponding to the different outcomes of interest in all participants.

While behavioral results point toward an opposite effect of the two used experimental conditions, we failed to detect distinctive changes specifically induced by extended practice with tasks not requiring exertion of impulse control. Indeed, while we observed tendencies toward an increased number of misses in the LCD condition, such differences did not reach significance relative to the HCD condition. This suggests that our statistical power could have been insufficient to appropriately detect and characterize these changes.

A higher number of participants or task trials could have been necessary to accurately identify behavioral changes in the LCD condition and their possible association with specific EEG signatures.

### Conclusions

Our results indicate that common EEG changes associated with task-dependent cognitive fatigue, such as the decreases in P300 and N200 ERP components, may not have a direct relationship with behavioral performance changes. Instead, we showed that the increase in commission errors and decreased reaction time followed by extended practice with tasks requiring impulse control are associated with the emergence of fast, automatic responses with distinct electrophysiological features. Specifically, such automatic responses are associated with ERPs characterized by a lack of recruiting frontal brain areas crucial for accurate response control and an incomplete categorization of the stimulus. We thus propose that fluctuations in the activation of task-related areas may underlie use-dependent behavioral alterations and contribute to the observed behavioral instability. Overall, our findings indicate that transient changes in neural activity may have a more important role than “stable” modulation in neural processing in shaping cognitive performance during extended task practice.

## Synthesis

Reviewing Editor: James Howard, Brandeis University Decisions are customarily a result of the Reviewing Editor and the peer reviewers coming together and discussing their recommendations until a consensus is reached. When revisions are invited, a fact-based synthesis statement explaining their decision and outlining what is needed to prepare a revision will be listed below. The following reviewer(s) agreed to reveal their identity: Michael Wiest. Note: If this manuscript was transferred from JNeurosci and a decision was made to accept the manuscript without peer review, a brief statement to this effect will instead be what is listed below.

This is an interesting study showing how cognitive load and fatigue from with-holding prepotent behavioral responses can affect subsequent performance on a go/nogo task that assesses stimulus discrimination and impulse control. The authors confirm previous results showing that cognitive fatigue induces more commission errors (aka false alarms), and further demonstrate the existence of a fast responding mode that sacrifices accuracy for speed. Importantly, the authors relate this behavioral response mode to underlying neural signatures in event-related potentials and spectra (reflecting synchronized rhythmic response components). As such, this is a significant contribution to our understanding of EEG signatures of cognitive control, which is medically and theoretically an important topic.

I do have some suggested corrections and clarifications. Because the lines in the submitted manuscript were not numbered, below I list my specific suggestions under the relevant section titles.

Abstract

"distinct kind of fast response" The word "response" is confusing here since it refers to pre-stimulus activity. I suggest something like "distinct neural signature on fast response trials, including..."

Experimental design

"with a classical response inhibition ^ (Go/NoGo, see below)" --> add missing word "task" at ^

Performance evaluation and statistical analyses

"calculated on hits (RT) post each session the respective value calculated during...": confusing, awkward.

ERPs analyses --> ERP analyses

"Subjects who did not exhibit discernible ERP responses...were excluded..." Please note how many subjects were excluded and from which conditions.

FT analysis

"Hamming window": what is the width of the window?

Results

"fraction of commission errors increased significantly" from what to what? Please report the absolute performance values.

Delta-%CE = 9 +- 11%. Is this 11% standard error or standard deviation? Since it appears to be a larger number than the SE in Figure 2, I infer that this is SD. Please confirm or correct, and similarly for all the other reported uncertainties.

Cortical activity underlying fast trials

"before the response" --> "before the behavioral button-press response"

"Instead, significant differences in the response-locked spectrum were found in theta...and alpha..." Please briefly say here which way the theta and alpha powers changed in the different conditions.

Figure 3 caption.

"(ERP) in cortical activity are associated with..." --> (ERPs) in cortical activity associated with..." I.e. add "s" and delete "are."

Cortical activity underlying commission errors in standard trials

"Commission errors are correlated with the fraction of FTs but do occur also in STs." I think it would be helpful to report the average fraction of CEs in FTs and STs.

Fronto-central event-related potentials in standard trials modulation by HCD and LCD

"according to previous studies focusing on the P300 (...), is localized..." Missing word.

"A significant change of P300 was observed..." Which direction was the change? What was its magnitude? Please report all effect sizes in this paragraph and throughout the paper. Also there is a missing period at the end of this paragraph.

Neural changes induced by extended cognitive load

Last paragraph of section: "...we observed a change in amplitude of P300..." --> Which way? Bigger or smaller?
