# Supplementary figures and images for: Extended Cognitive Load Induces Fast Neural Responses Leading to Commission Errors
Source: eNeuro. 2025 Feb 6;12(2):ENEURO.0354-24.2024. doi: 10.1523/ENEURO.0354-24.2024 (PMC11810548; doi:10.1523/ENEURO.0354-24.2024)

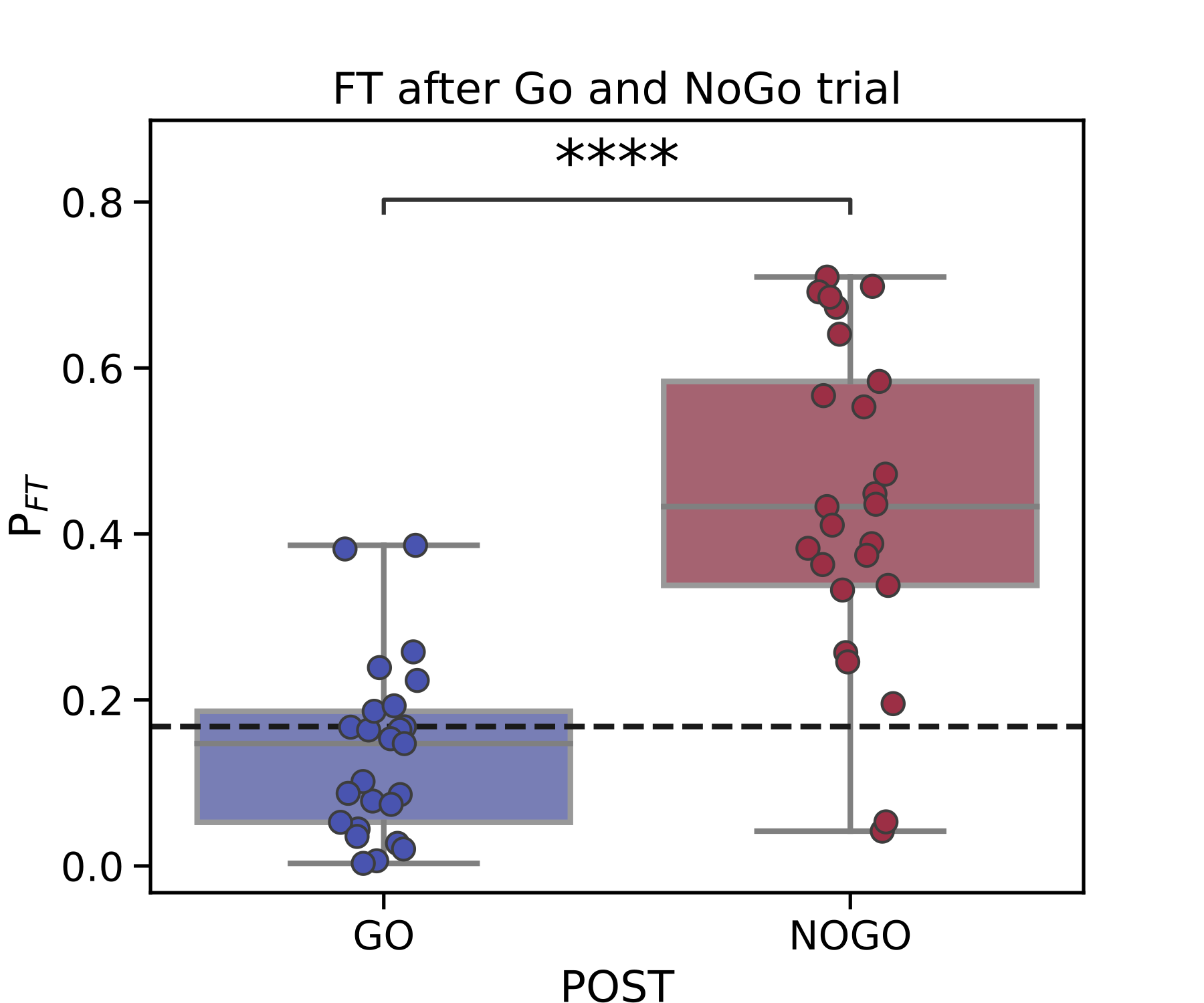

Supplement: Figure 2-1 — Probability of FT occurrence after a Go or NoGo trial calculated on all trials for each subject (p < 0.00001, Wilcoxon Test). Download Figure 2-1, TIF file. [file eneuro-12-ENEURO.0354-24.2024-s002.tif]

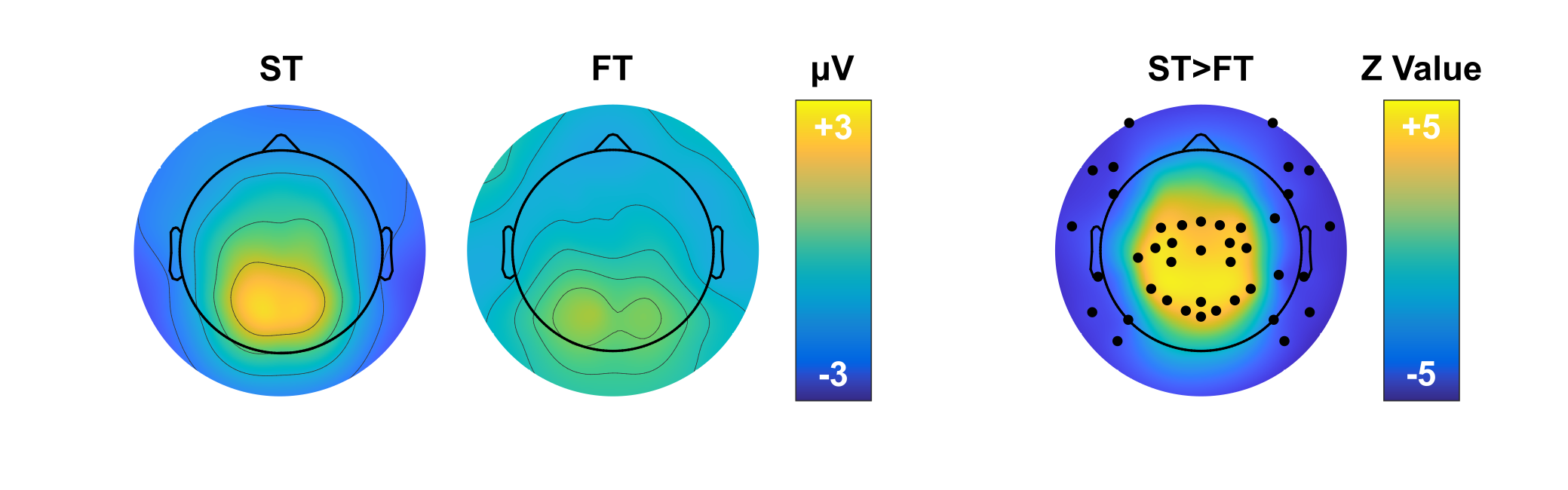

Supplement: Figure 3-1 — Comparison of the potential distribution on the scalp between FTs and STs in the 150 ms preceding the response. Black dots represent the electrodes for which a significant difference has been found (Wilcoxon test, FDR correction). Download Figure 3-1, TIF file. [file eneuro-12-ENEURO.0354-24.2024-s003.tif]

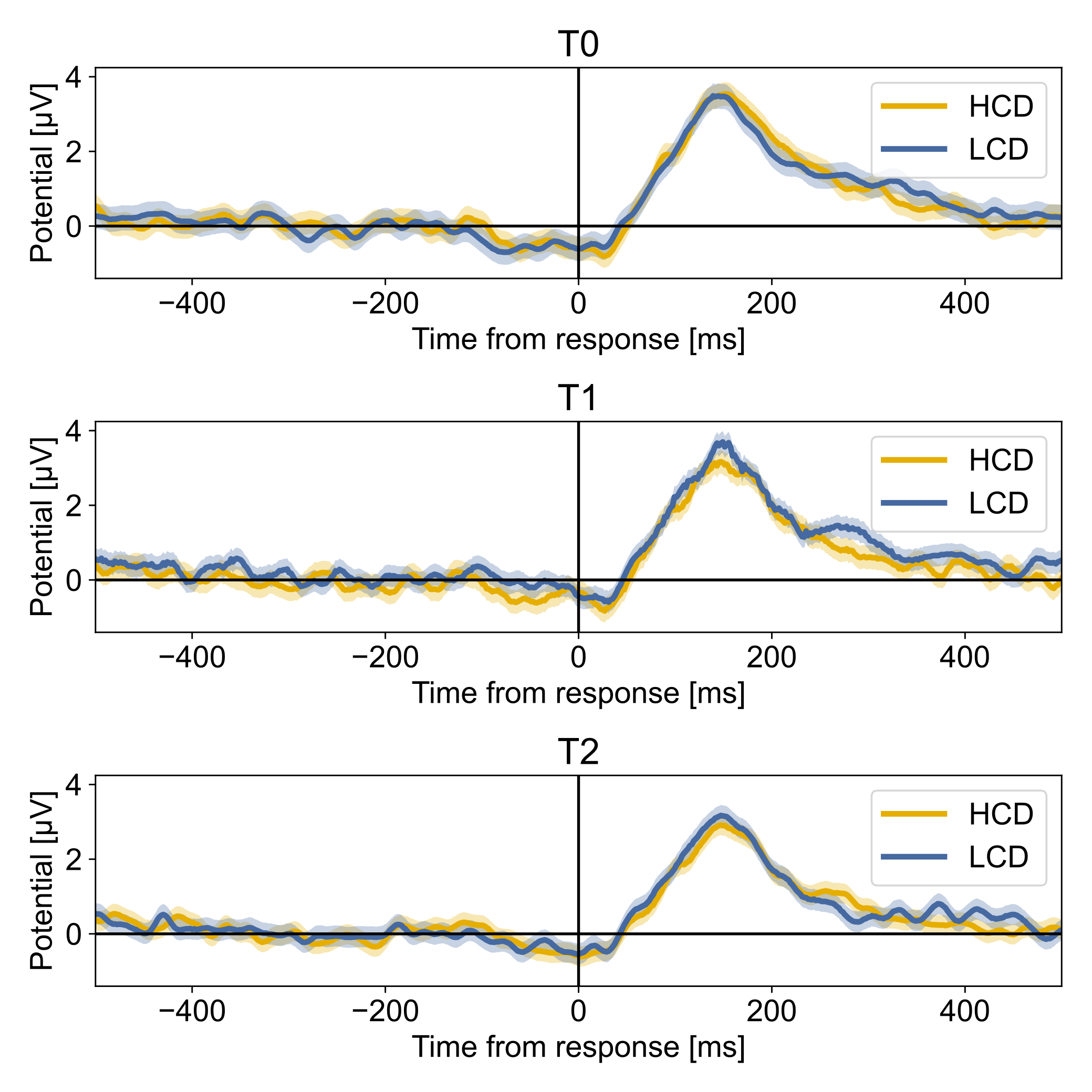

Supplement: Figure 3-2 — Between sessions differences in anterior ROI for FTs response-locked epochs. The differences are assessed in the same way in Figure 3A, B, D, E. Download Figure 3-2, TIF file. [file eneuro-12-ENEURO.0354-24.2024-s004.tif]

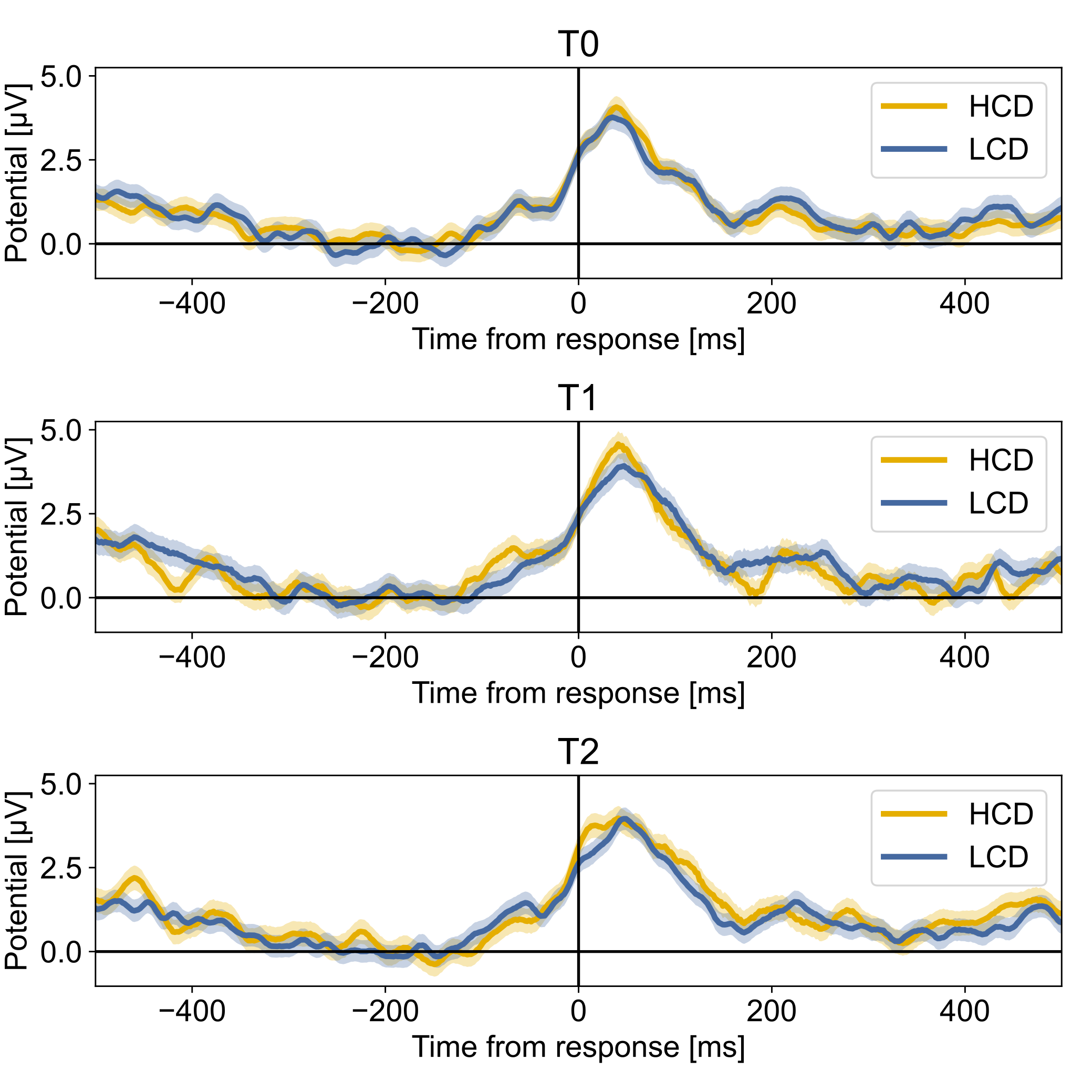

Supplement: Figure 3-3 — Between sessions differences in posterior ROI for FTs response-locked epochs. The differences are assessed in the same way in Figure 3A, B, D, E. Download Figure 3-3, TIF file. [file eneuro-12-ENEURO.0354-24.2024-s005.tif]

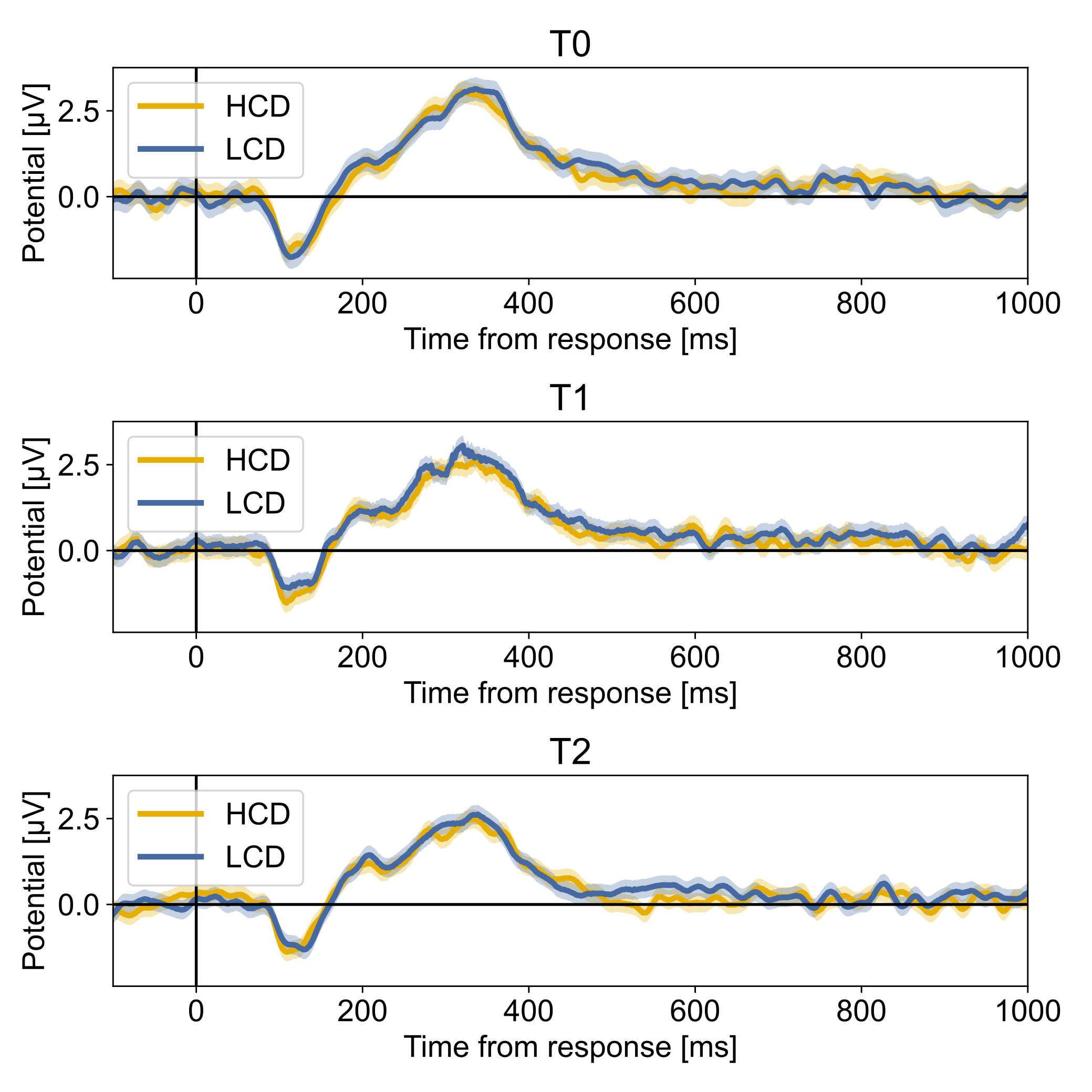

Supplement: Figure 3-4 — Between sessions differences in anterior ROI for FTs stimulus-locked epochs. The differences are assessed in the same way in Figure 3A, B, D, E. Download Figure 3-4, TIF file. [file eneuro-12-ENEURO.0354-24.2024-s006.tif]

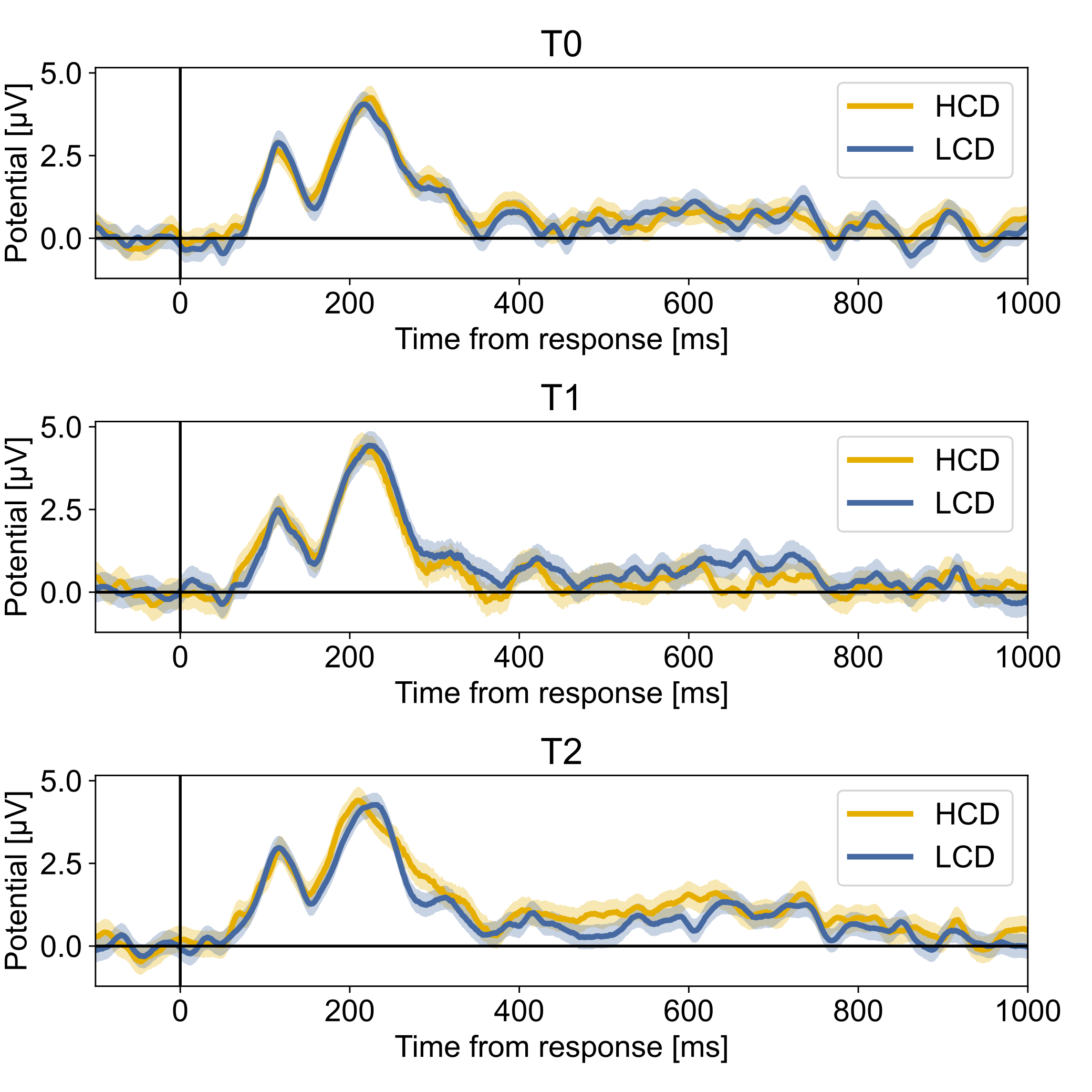

Supplement: Figure 3-5 — Between sessions differences in posterior ROI for FTs stimulus-locked epochs. The differences are assessed in the same way in Figure 3A, B, D, E. Download Figure 3-5, TIF file. [file eneuro-12-ENEURO.0354-24.2024-s007.tif]

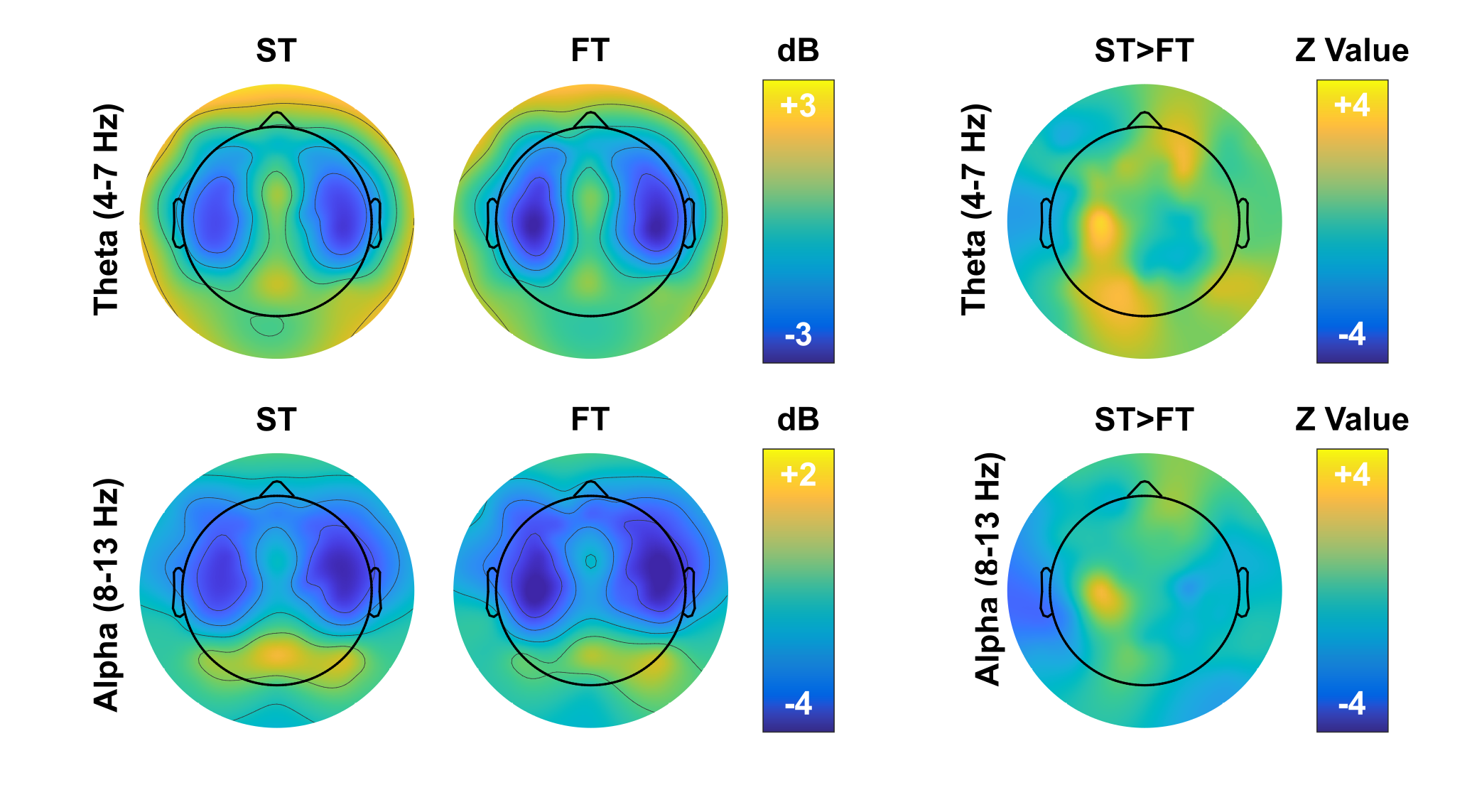

Supplement: Figure 3-6 — Theta and Alpha band FT-ST pre-stimulus [-300,0]. Black dots represent the electrodes for which a significant difference has been found as in Figure 3 F, G (p < 0.05, Wilcoxon test, FDR correction). Download Figure 3-6, TIF file. [file eneuro-12-ENEURO.0354-24.2024-s008.tif]

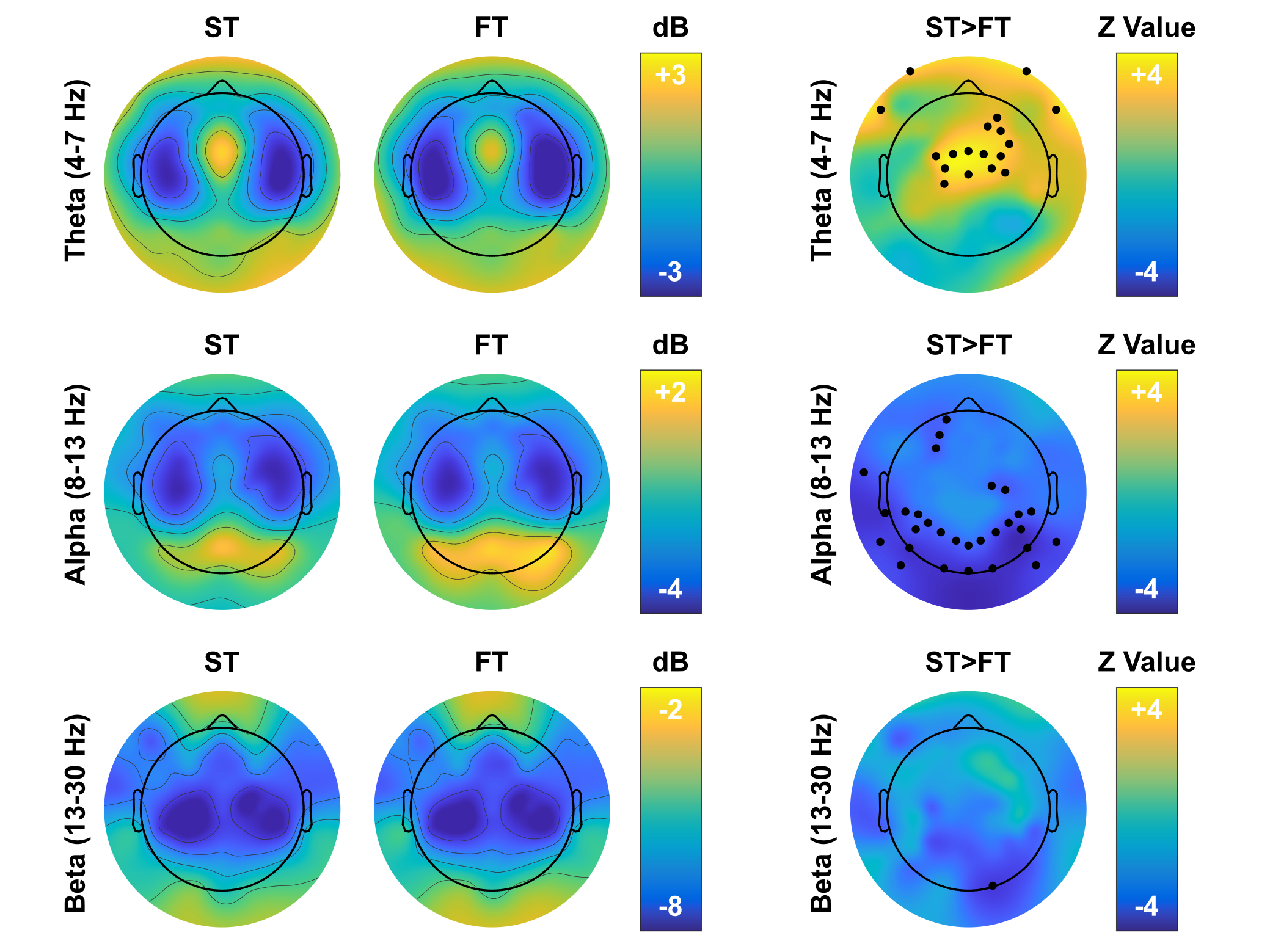

Supplement: Figure 3-7 — Theta, alpha, and Beta band power difference between ST and FT in response-locked. Black dots represent the electrodes for which a significant difference has been found as in Figure 3 F, G (p < 0.05,Wilcoxon test, FDR correction). Download Figure 3-7, TIF file. [file eneuro-12-ENEURO.0354-24.2024-s009.tif]
